# Effects of Excessive Activation of N-methyl-D-aspartic Acid Receptors in Neonatal Cardiac Mitochondrial Dysfunction Induced by Intrauterine Hypoxia

**DOI:** 10.3389/fcvm.2022.837142

**Published:** 2022-03-30

**Authors:** Yang Liu, Ziqiang Luo, Zhengchang Liao, Mingjie Wang, Yan Zhou, Siwei Luo, Ying Ding, Teng Liu, Chuangding Cao, Shaojie Yue

**Affiliations:** ^1^Department of Neonatology, Xiangya Hospital, Central South University, Changsha, China; ^2^Department of Physiology, Xiangya School of Medicine, Central South University, Changsha, China; ^3^Departments of Pediatrics and Neonatology, Children's Hospital of Fudan University, Shanghai, China; ^4^Laboratory of Neonatal Diseases, National Children's Medical Center, National Commission of Health, Shanghai, China

**Keywords:** intrauterine hypoxia, N-methyl-D-aspartic acid receptors, mitochondrial dysfunction, neonatal rat heart, mitochondrial dynamics

## Abstract

Intrauterine hypoxia is a common complication during pregnancy and could increase the risk of cardiovascular disease in offspring. However, the underlying mechanism is controversial. Memantine, an NMDA receptor antagonist, is reported to be a potential cardio-protective agent. We hypothesized that antenatal memantine treatment could prevent heart injury in neonatal offspring exposed to intrauterine hypoxia. Pregnant rats were exposed to gestational hypoxia or antenatal memantine treatment during late pregnancy. Newborns were then sacrificed to assess multiple parameters. The results revealed that Intrauterine hypoxia resulted in declining birth weight, heart weight, and an abnormally high heart weight/birth weight ratio. Furthermore, intrauterine hypoxia caused mitochondrial structural, functional abnormalities and decreased expression of DRP1, and upregulation of NMDAR1 *in vivo*. Antenatal memantine treatment,an NMDARs antagonist, improved these changes. *In vitro*, hypoxia increased the glutamate concentration and expression of NMDAR1. NMDAR activation may lead to similar changes in mitochondrial function, structure, and downregulation of DRP1 *in vitro*. Pharmacological blockade of NMDARs by the non-competitive NMDA antagonist MK-801 or knockdown of the glutamate receptor NR1 significantly attenuated the increased mitochondrial reactive oxygen species and calcium overload-induced by hypoxia exposure. These facts suggest that memantine could provide a novel and promising treatment for clinical use in intrauterine hypoxia during pregnancy to protect the cardiac mitochondrial function in the offspring. To our best knowledge, our research is the first study that shows intrauterine hypoxia can excessively activate cardiac NMDARs and thus cause mitochondrial dysfunction.

## 1. Introduction

Intrauterine hypoxia (IUH) is a common complication of pregnancy that can be caused by maternal anemia, preeclampsia, intrahepatic cholestasis, and antenatal stress. Our team previously reported that IUH could result in long-lasting damage in the brain of offspring (1). Increasing evidence validated the theory of fetal origins of adult disease and that development of cardiovascular disease in adulthood might be associated with intrauterine insults (2–4). A study in rats revealed that antenatal hypoxia could also increase the severity of heart damage after ischemia-reperfusion injury in offspring (5). However, the underlying mechanism of the pathogenesis of impaired cardiovascular function is still controversial and unclear.

A previous study found that hypoxia during pregnancy interfered with mitochondrial fusion and fission in the neonatal rat heart, which led to mitochondrial ultrastructural damage and decreased mitochondrial respiratory function. It might imply that the mitochondrial dynamics is a possible target of the antenatal hypoxia-induced heart injury of the newborn rat (6). Normally functioning mitochondrial dynamics would remove unhealthy mitochondria and therefore maintain mitochondrial homeostasis. The high demand for energy metabolism in the myocardium increased the risk of cardiovascular disease in patients with mitochondrial disorders (7). Thus, mitochondrial dysfunction has been associated with numerous cardiovascular diseases (8).

N-Methyl-D-aspartate receptors (NMDARs) have been widely investigated in the central nervous system. Overactivation of NMDAR was associated with the excitotoxicity of neurons. Research showed that NMDARs also existed in peripheral tissues such as lung, liver, kidney, and heart (9). Our group also demonstrated that excessive expression of NMDAR might be related to prenatal hypoxia-induced impaired pancreatic function in offspring (10). The function of the NMDAR in heart tissue remains elusive. Previous studies demonstrated that NMDAR played an essential role in heart development, cardiac electrophysiology, and myocardial fibrosis (11, 12). Sun reported that ischemia-reperfusion-induced ventricular arrhythmias in rats were significantly improved by MK-801(a noncompetitive NMDA receptor antagonist) (13). Memantine, an NMDA receptor antagonist, was shown to ameliorate the heart remodeling, lipid peroxidation, and neutrophil infiltration (14). NMDA-induced excitotoxicity in cerebellar granule neurons led to mitochondrial morphology as evidenced by the presence of fragmented mitochondria, cessation of mitochondrial fusion, and cristae dilation (15). However, the exact role of NMDAR in IUH-induced heart injury and the relationship between NMDAR and mitochondrial dynamics are still unclear. Considering the existence of cardiac NMDARs and the potential protective effect of memantine, we hypothesized that IUH-induced mitochondrial dysfunction in neonatal rat hearts is mediated by the excessive activation of NMDA receptors.

## 2. Materials and Methods

### 2.1. Animals

Female (200–250 g) and male (300–350 g) Sprague-Dawley (SD) rats were provided by the Animal Center of Central South University (CSU), Changsha, China. The rats were housed, acclimatized, and mated in the Animal Center of CSU. Day 0 of gestation (G0) was determined by examining the female rats for the presence of vaginal plugs. The pregnant rats were randomized into four groups (*N* = 4/group),and the detailed group information was provided in the Supplementary Table 1. At gestation day 19–20 (G19-20), the pregnant rats received one of four treatments as follows: (1) rats in the control group were maintained under normoxic conditions at 21% O_2_ (Air) at G19-20; (2) rats were maintained in normoxic conditions and received daily intraperitoneal injections of memantine (Sigma, USA) at 5 mg/kg body weight per day at G19-20 (Air plus memantine); (3) rats were placed in a hypoxic environment with FiO2 at 9.5–11.5% for 8 h per day at G19-20, as was previously published (16) (Hypoxia). The FiO2 of the pregnant rats was checked every 30 min to ensure the stable hypoxia environment *in vivo*; and (4) rats were treated with memantine (5 mg/kg body weight) intraperitoneally 30 min before the hypoxia exposure (Hypoxia plus memantine). Both the hypoxia exposure and memantine administration occurred at 8 a.m. on G19 and G20. After natural delivery, all the newborn rats were weighed and sacrificed immediately. Heart tissue was isolated and weighed.

### 2.2. Hypoxic Model *in vitro*

H9c2 cardiomyoblast cells were cultured in Dulbecco's modified Eagle's medium (DMEM) (Hyclone, Thermo Scientific) supplemented with 10% fetal bovine serum (Foundation, Gemini, South America). Cells were incubated in a humidified incubator with 5% CO_2_ at 37°C. The media was changed every 2 days, and the cells were used in the logarithmic growth phase. Hypoxia treatment of H9c2 cells at 10% O_2_ mimicked developmental hypoxia *in vitro* and was conducted as described previously (17). Cultured H9C2 cells were randomly assigned into three groups:(1) control group (Con), cells cultured under standard incubation conditions; (2) Hypoxia 24 h group, cells placed in a hypoxic chamber for 24 h; (3) Hypoxia 48 h group, cells placed in a hypoxic chamber for 48 h. The oxygen concentration was monitored by an automatic hypoxia chamber (shenzhengkuyuan, MIC-101).

### 2.3. Transmission Electron Microscopy

Transmission electron microscopy was performed as previously published (6). Cardiac tissues (about 1 mm^3^ pieces) were obtained from the different groups and cell culture samples. The specimens were then washed, dehydrated, embedded in resin, stained with uranyl acetate and lead nitrate, and sectioned into 50–70 nm Ultrathin sections. The sections were examined under a Hitachi electron microscope (H600; Hitachi, Tokyo, Japan). ImageJ software was used to obtain two well-characterized indices of mitochondrial fission and fusion, the mitochondrial interconnectivity (ratio of area to the perimeter) and mitochondrial elongation (ratio of the lengths of major and minor axes) as previously described (18, 19). Measurements were performed and averaged for 50 randomly selected mitochondria per group by an examiner blinded to the group information. The increased mitochondrial elongation and interconnectivity indicated reduced mitochondrial fragmentation.

### 2.4. Wheat Germ Agglutinin Staining

The assessment of the cardiomyocytes area was assessed by wheat germ agglutinin (WGA) staining. Briefly, heart tissue slides were stained at room temperature for 30 min with WGA-FITC(fluorescein isothiocyanate) labeled antibody (1:40, W11262, Invitrogen).

### 2.5. Citrate Synthase Activity Assay

According to the manufacturer's protocol, citrate synthase (CS) activity was measured using a kit (Catalog no. MAK193, Sigma, St. Louis, MO). Briefly, heart tissue samples were used for each enzymatic analysis. Measurements were performed in triplicate for each experimental condition) at room temperature in 96 well dishes. The enzyme was immunocaptured within the microplate wells, and activity was determined by measuring the color development of TNB, which is generated in the reaction of citrate synthesis. Enzyme activity was measured spectrophotometrically (at an absorbance wavelength of 412 nm). The formula provided by the manufacturer was used to calculate total CS activity.

### 2.6. ATP Concentration Measurement

According to the manufacturer, ATP concentration was determined by Phosphomolybdic acid colorimetry using an ATP assay kit (Catalog no. A095, Nanjing jiancheng, China) on a Thermo Forma plate reader's protocol.

### 2.7. Reactive Oxygen Species and MitoROS Measurement

Reactive oxygen species (ROS) levels in neonatal heart tissue were measured by enzyme-linked immunosorbent assay (ELISA) based on the manufacturer's instructions (Duma biological company, Shanghai, China). The ROS fluorescent probe (DCFH-DA, Beyotime, Shanghai, China) and MitoSOX Red (ThermoFisher) were used to detect ROS and mitoROS levels in the fixed H9c2 cells according to the manufacturer's protocol.

### 2.8. Mitochondrial DNA Copy Number Analysis

Quantitative polymerase chain reaction (qPCR) was used to determine mitochondrial DNA copy number as previously described (20). According to the manufacturer's protocol, DNA was extracted from tissues using a DNA extraction kit (Catalog no. DP304-03, TIANGEN, Beijing, China). Hexokinase 2 (HK2) and NADH dehydrogenase subunit 1 (ND1) were chosen as representative examples of a nuclear-encoded gene and a mitochondrial encoded gene, respectively. Comparison of ND1 expression relative to HK2 expression represented the mtDNA copy number/ nDNA ratio.

### 2.9. Glutamate Assay

The concentration of glutamate in the medium was determined using a colorimetric assay kit according to the manufacturer's protocol (Abcam, Toronto, ON, Canada, ab83389).

### 2.10. Cell Viability Assay

According to the manufacturer's protocol, a cell counting kit (CCK-8/WST-8) was used to measure cell viability (Dojindo, Japan). Cell viability was monitored at a wavelength of 450 nm with a Bio-Tek Synergy H1 microplate reader (Winooski, VT, USA).

### 2.11. JC-1 Assay for Mitochondrial Membrane Potential

Treated H9c2 cells were stained with the membrane-permeant JC-1 dye (Beyotime, Shanghai, China) to assess mitochondrial membrane potential (MMP). Cells were treated with NMDA (1 and 3 mM) for 24 h, digested by trypsin, and then washed with phosphate-buffered saline (PBS). The harvested cell samples were stained with JC-1 (10 μg/ml) for 20 min in the cell incubator at 37. After JC-1incubation, samples were centrifuged at 600 g (4) for 3–4 min and washed with PBS. The cells were analyzed using a flow cytometer (Beckman,A00-1-1102). Fluorescent signals for J-aggregates (Red) and J-monomers (Green) were read at excitation and emission wavelengths of 535 and 595 nm and 485 and 535 nm, respectively. The red/green fluorescence percentage ratio was then calculated to estimate the mitochondrial potential membrane.

### 2.12. Intracellular Calcium Level Assay

According to the manufacturer's guide, the manufacturer's guide measured Intracellular calcium levels using the Fluo-3 AM fluorescent probe (Beyotime, Shanghai, China). Fluo-3 AM is a fluorescent dye that enters the cell membrane, where it is cleaved by esterase into Fluo-3, which combines with Ca2+. Briefly, the Medium was removed, and cells were incubated with the Fluo-3 AM (5 μm) for 30 min at room temperature. Then the Fluo-3 AM was then removed, and the intensity of intracellular calcium fluorescence was detected by fluorescence microscopy as previously described (21). The calcium concentration is based on the mean fluorescence intensity.

### 2.13. Quantitative Reverse Transcriptase Polymerase Chain Reaction

Quantitative reverse transcriptase-polymerase chain reaction (qRT-PCR) was conducted as previously published (6). Primer information is provided in Supplementary Table 2.

### 2.14. Western Blot Assay

According to the manufacturer's manufacturer, the protein from cardiac tissue and H9c2 cells were extracted using radioimmunoprecipitation assay (RIPA) lysis buffer (P0013B, Beyotime, Shanghai, China) protocol. SDS-PAGE (10%) was used to electrophoretically separate proteins followed by transfer to polyvinylidene fluoride (PVDF) membranes (Merck, Millipore, Billerica, MA, USA). Subsequently, the membranes were blocked with 5% non-fat milk in Tris-buffered saline with Tween 20 (TBST) for 1 h at room temperature, and incubated with the following primary antibodies: anti-dynamin related protein 1 (DRP1) (1:1,000), anti-NMDAR1 (1:200) purchased from Abcam, and β−actin (1:5,000) and anti-BNP (1:500) purchased from Proteintech. Samples incubated overnight at 4°C with primary antibodies and incubated with horseradish peroxidase (HRP)-labeled goat anti-rabbit or HRP-labeled goat anti-mouse IgG secondary antibodies (Proteintech) at a dilution of 1:5,000. Ultrasensitive enhanced chemiluminescent solution (Proteintech) was used for detection and quantified using a FluorChem Chemiluminescence Imaging System (ProteinSimple, San Jose, CA) via densitometry.

### 2.15. Knockdown of NMDAR1 (NR1) Using RNA Interference

H9c2 cells were transfected in serum-free media using LipofectamineTM2000 according to the manufacturer's protocol. Short-interfering RNA (siRNA) directed against NR1 was purchased from Honorgene. The gene knockdown efficiency was evaluated by western blotting (Supplementary Figure 1).

### 2.16. Statistical Analysis

The data were analyzed by one-way ANOVA followed by Tukey's test or Student's *t*-test. *P* < 0.05 was considered to be statistically significant.

## 3. Results

### 3.1. NMDAR Subunits Expression in the Heart

**Figure 1:**
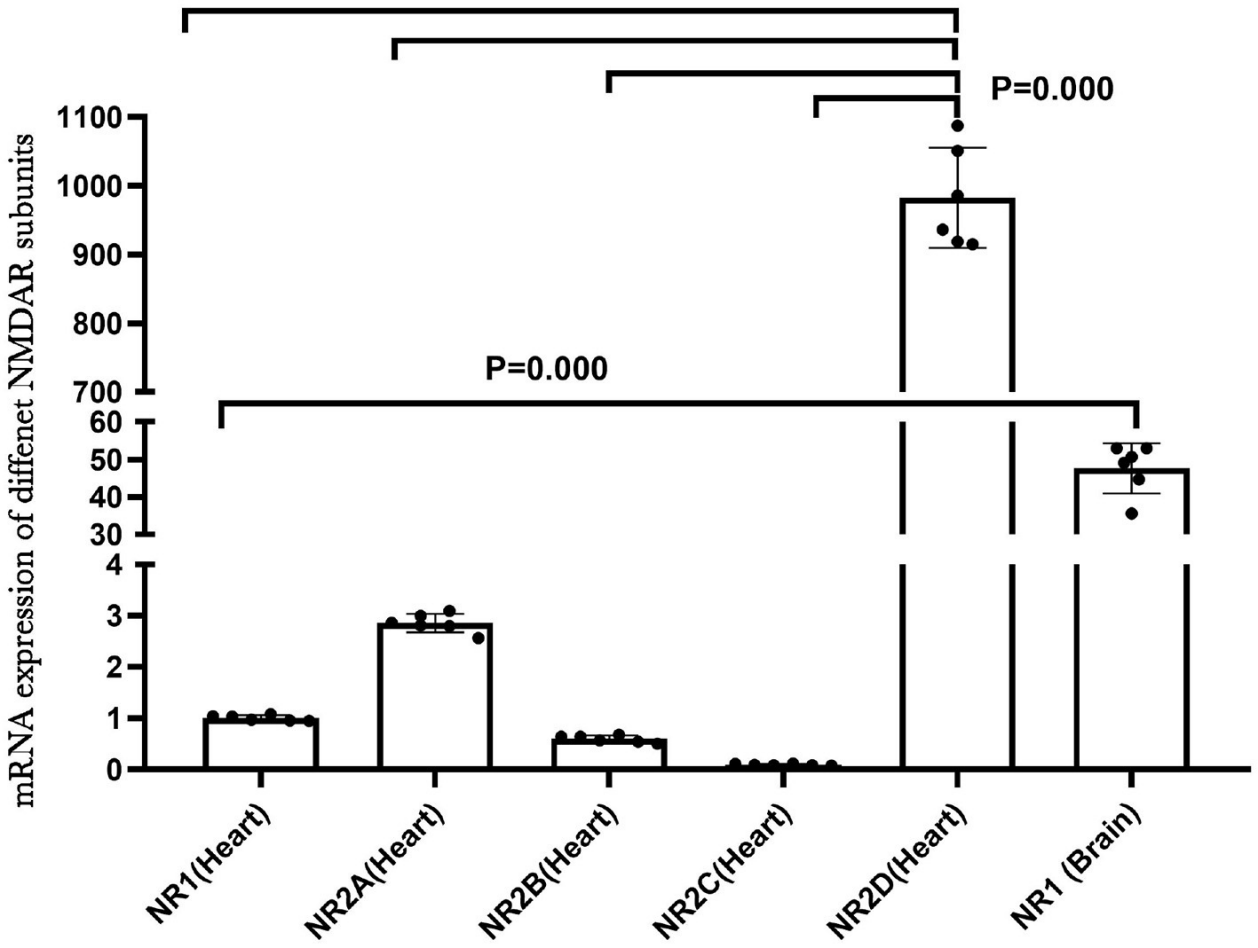
NMDA receptor subunits expression in the neonatal rat heart.

NMDA receptors are abundant and well-studied in the central nervous system. To understand the role of NMDAR in the heart, we examined the mRNA expression of different NMDAR subunits in neonatal rat hearts by qRT-PCR. The results indicated that the multiple NMDAR subunits were expressed in neonatal rat hearts and that NR2D showed the highest expression level. We also compared the mRNA expression of the NR1 subunit in the brain and heart, and the NR1 expression was much higher in the brain (*P* < 0.01; Figure 1). In addition, the immunofluorescence results of NR1 (red) and CTnT (a marker of cardiomyocytes, green) in the newborn rat heart validated the existence of NR1 in the neonatal cardiomyocytes (Supplementary Figure 2).

### 3.2. Antenatal Memantine Treatment Decreased the Up-Regulation of NR1 in the Neonatal Rat Heart Induced by the Intrauterine Hypoxia

**Figure 2:**
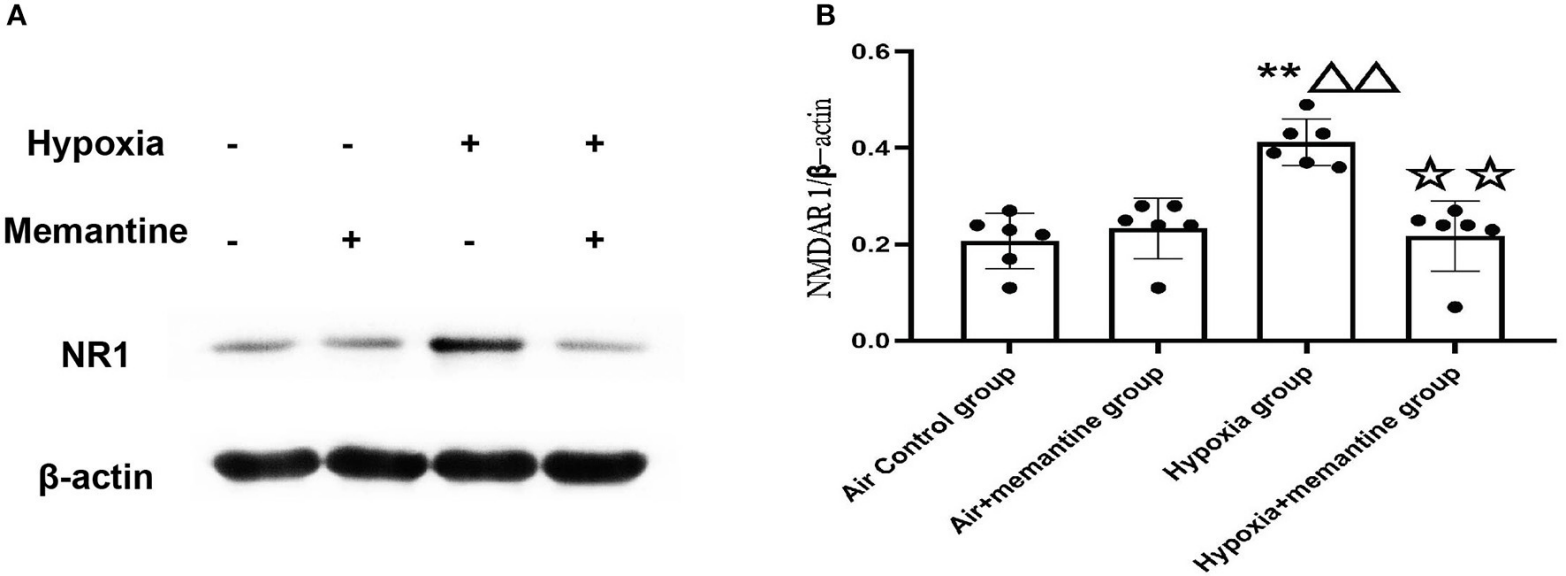
Antenatal memantine treatment reduced the intrauterine hypoxia elevated NR1 protein expression in newborn rat heart. The representative western blot images of NR1 **(A)** and semi-quantified level of NR1 **(B)**. Compared with the air control group: ^*^P < 0.05,^**^P < 0.01; compared with the air+memantine group: ^▵^*P* < 0.05,^▵▵^*P* < 0.01; compared with the hypoxia group: ^✰^*P* < 0.05,^✰✰^*P* < 0.01. All data are presented as a mean ± S.E.M. *N* = 6. All blot images are representative.

NMDA receptors are heterotetrameric assemblies of subunits, including NR1 and NR2 (2A-D). Classic NMDARs consist of two indispensable NR1 and two NR2. The NR1 subunit is essential for channel activity, whereas the NR2 subunits can modulate functional diversity of NMDARs (22). Thus, we explored the expression of NR1 under intrauterine hypoxia exposure. NR1 expression was examined by western blotting. The data showed that the hypoxic insult during pregnancy increased the expression of the NR1 protein (Figure 2). In the Hypoxia+memantine group, the NR1 expression significantly decreased compared to the hypoxia group. Only memantine did not change the expression of NR1. The mRNA expression of the NR2D in the animal model was also examined. The results showed that the antenatal hypoxia insult could significantly increase the expression of NR2D (*P* < 0.05; Supplementary Figure 3).

### 3.3. Antenatal Memantine Treatment Improved the Low Rat Birth Weight, Heart Weight and Decreased the Ratio of Heart Weight/ Birth Weight Induced by Intrauterine Hypoxia

**Figure 3:**
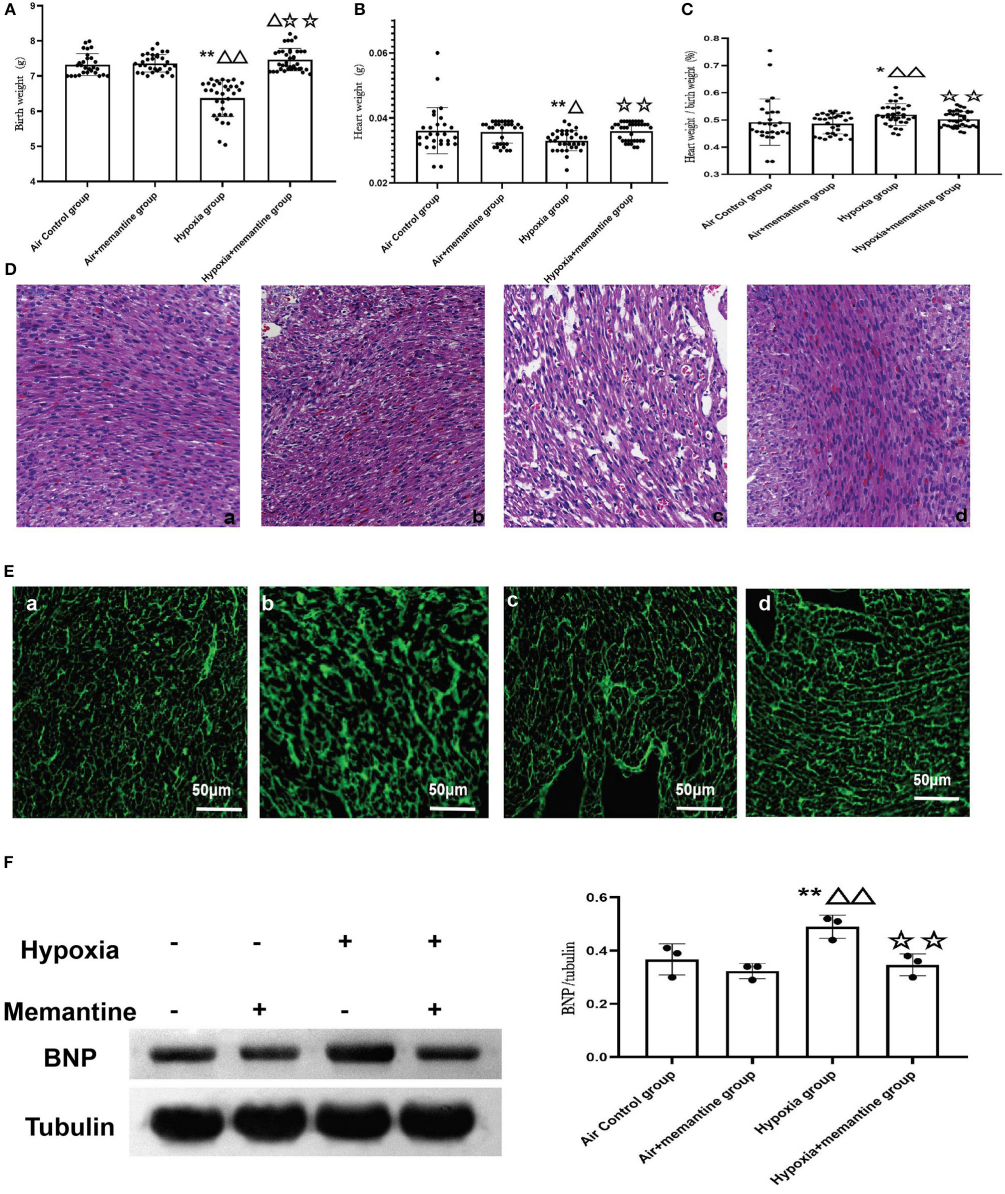
**(A–D)** Antenatal memantine treatment improved low rat birth weight **(A)**, heart weight **(B)** and decreased the ratio of heart weight/ birth weight **(C)** induced by intrauterine hypoxia. **(D)** Representative images of HE staining (200x). (a): air control group; (b): air + memantine group; (c): hypoxia group; (d): hypoxia + memantine group. **(E)** Representative images of WGA staining (400x). (a): air control group; (b): air + memantine group; (c): hypoxia group; (d): hypoxia + memantine group. **(F)** The representative western blot images of BNP (a) and semi-quantified level of BNP (b). Compared with the air control group: ^*^P < 0.05,^**^P < 0.01; compared with the air+memantine group: ^▵^*P* < 0.05,^▵▵^*P* < 0.01; compared with the hypoxia group: ^✰^*P* < 0.05,^✰✰^*P* < 0.01. *N* = 28 for air control group; *N* = 31 for air + memantine group; *N* = 33 for hypoxia group; *N* = 37 for hypoxia + memantine group.

Memantine is a commonly used NMDAR antagonist. We then examined the effects of memantine treatment in pregnant rats exposed to hypoxia. Compared to the control animals, hypoxia during late pregnancy (G19-20) significantly decreased the birth weight (BW) of the offspring (*P* < 0.01). Antenatal memantine treatment significantly alleviated the BW reduction of the offspring born to hypoxia-exposed rats (Figure 3A, *P* < 0.01). The memantine-treated air control group had a similar birth weight to the air control group indicating little toxicity of the memantine. The heart weight (HW) data demonstrated a similar trend to BW in that hypoxia decreased HW in the offspring of hypoxia-treated animals compared to the controls, and memantine treatment improved HW (Figure 3B, *P* < 0.01).

IUH also increased the ratio of heart weight/birth weight (Figure 3C, *P* < 0.05), and antenatal memantine treatment effectively reduced the abnormally high ratio of heart weight/birth weight (Figure 3C, *P* < 0.01). No effect of memantine was seen in the air+memantine group (Figure 3C).

We examined the heart tissue by histological staining. Compared with normal hearts, neonatal offspring hearts exposed to IUH showed loosely arranged cardiac muscle fibers and increased interstitial distances as evaluated by hematoxylin and eosin (H&E) staining (Figure 3Dc). However, hearts in the hypoxia+memantine group maintained normal myocardial histological structures comparable to those of control hearts (Figure 3Dd). WGA staining was used to evaluate the cell surface area of the cardiomyocytes (Figure 3E). The hypoxia group showed a significantly larger surface of the cardiomyocytes. In addition, we explored the BNP protein expression in the animal model as a marker of cardiac hypertrophy. WB analysis demonstrated that the IUH significantly increased the expression of BNP (Figure 3F).

### 3.4. Antenatal Memantine Treatment Attenuated Neonatal Rat Cardiac Mitochondrial Ultrastructural Damage and Dysfunction Induced by Intrauterine Hypoxia

**Figure 4:**
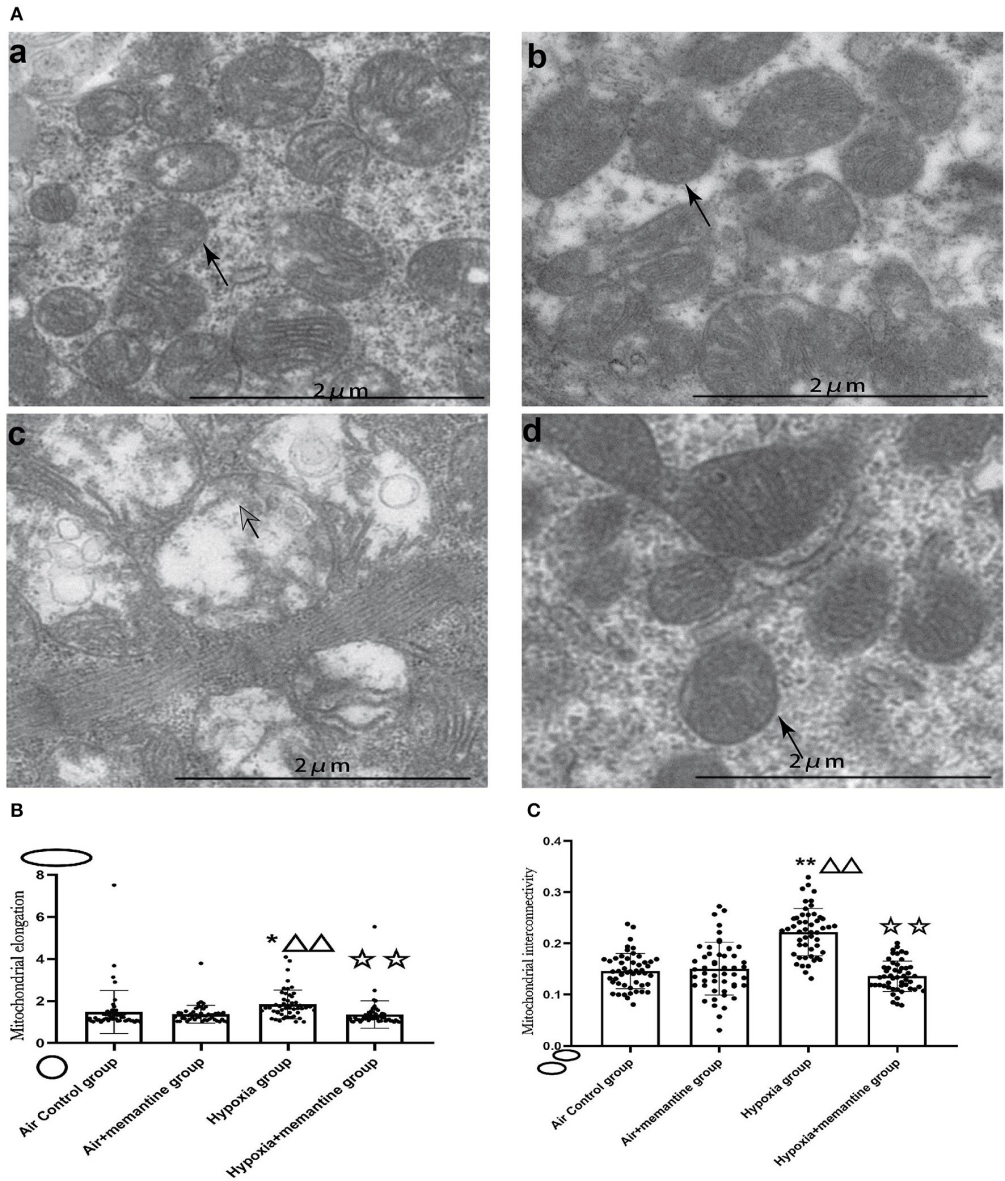
Antenatal Memantine treatment attenuated the neonatal rat cardiac mitochondrial ultrastructural damage induced by intrauterine hypoxia. **(A)** Representative images of TEM (5000X). (a): Air control group; (b). Air+memantine group; (c). Hypoxia group; (d). Hypoxia+memantine group;solid arrows: normal mitochondria; hollowed arrows: damaged mitochondria. **(B)** Mitochondrial elongation. **(C)** Mitochondrial interconnectivity. Compared with the air control group: ^*^P < 0.05,^**^P < 0.01; compared with the air+memantine group: ^▵^*P* < 0.05,^▵▵^*P* < 0.01; compared with the hypoxia group: ^✰^*P* < 0.05,^✰✰^, *P* < 0.01. All data are presented as mean ± S.E.M.

We examined the ultrastructure of mitochondria in neonatal rat hearts in offspring of animals exposed to air or IUH. The air control group had healthy mitochondria characterized by compact matrix cristae and a continuous outer membrane (Figure 4Aa). In contrast, the hypoxia group had damaged mitochondria characterized by fragmented cristae, a ballooned matrix, and a ruptured outer membrane (Figure 4Ac). After memantine treatment, the damaged mitochondria appeared normal (Figure 4Ad). Additionally, we quantified the mitochondrial morphology by analyzing each group's mitochondrial elongation and interconnectivity. The data revealed that the hypoxia group had an increased average elongation and interconnectivity and antenatal memantine treatment decreased the high levels of elongation and interconnectivity after IUH (Figures 4B,C, *P* < 0.01). These results suggested that mitochondrial fragmentation was reduced in the hypoxia group. These results indicated that mitochondrial fission was reduced or mitochondrial fusion increased in the hypoxia group.

**Figure 5:**
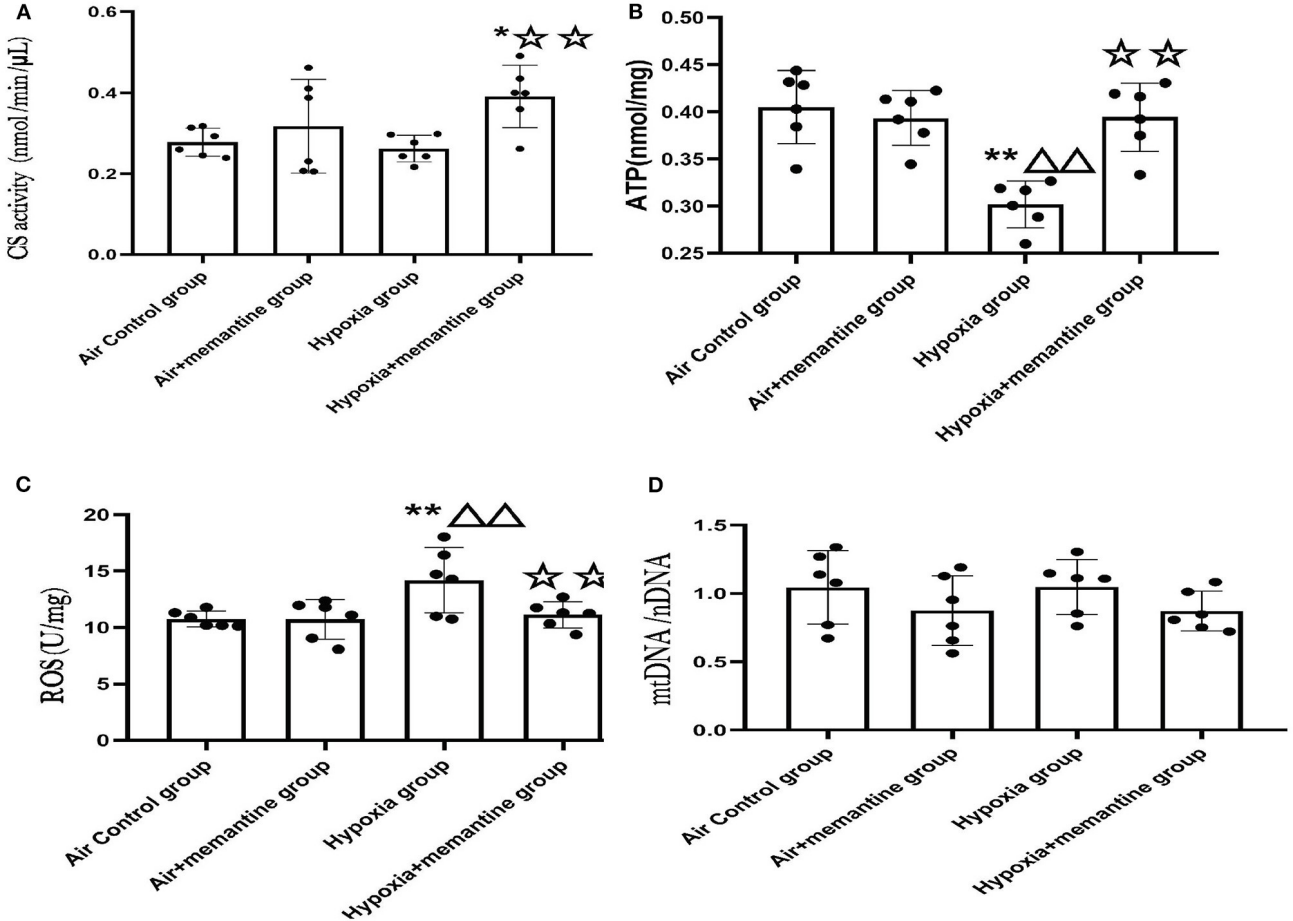
Antenatal memantine treatment attenuated the mitochondrial dysfunction in neonatal rat heart induced by intrauterine hypoxia. **(A)** CS activity in different groups (nmol/min/μL); **(B)** ATP concentration in different groups (nmol/mg); **(C)** ROS levels in different groups (U/mg); **(D)** mtDNA copy number (mtDNA/nDNA) in different groups. Compared with the air control group: ^*^P < 0.0;^**^P < 0.01; ^▵^*P* < 0.05,^▵▵^*P* < 0.01; compared with the air+memantine group: ^▵^*P* < 0.05,^▵▵^*P* < 0.01; compared with the hypoxia group: ^✰^*P* < 0.05,^✰✰^*P* < 0.01. All data are presented as mean ± S.E.M. *N* = 6.

To analyze cardiac mitochondrial content and function in the neonatal rat heart, we investigate citrate synthase (CS) activity, adenosine triphosphate (ATP) concentration. Reactive oxygen species (ROS) levels and mitochondrial copy number (Figure 5).

CS, the first rate-limiting enzyme of the tricarboxylic acid cycle, is a mitochondrial enzyme found in the mitochondrial matrix and is routinely used as a marker of mitochondrial content (23). The hypoxia group showed a slight decline in CS activity compared to the control group, although it was not statistically significant. The memantine + hypoxia group was significantly higher when compared with the air control group and the hypoxia group (Figure 5A, *P* < 0.05 or 0.01). Like other results such as ATP and ROS, memantine treatment did not affect the air+memantine group (Figure 5A).

As ATP is formed in mitochondria, decreased ATP levels represent mitochondrial dysfunction in many cases. We examined ATP levels in the neonatal rat hearts. The ATP concentration of the hypoxia group was significantly lower than the air control group (Figure 5B, *P* < 0.01). Antenatal memantine treatment improved ATP production in the memantine+hypoxia group (Figure 5B, *P* < 0.01). Additionally, memantine treatment did not affect the air+memantine group (Figure 5B).

Impairment of mitochondrial function often results in excessive ROS production and subsequent oxidative stress. Excessive ROS levels can also act as a risk factor for mitochondrial damage. We measured ROS levels in the neonatal hearts tissue. The hypoxia group's ROS level was significantly increased compared with the air control group (Figure 5C, *P* < 0.01). Antenatal memantine treatment decreased ROS levels in the memantine+hypoxia group (Figure 5C, *P* < 0.01). Additionally, the memantine treatment did not affect the air +memantine group (Figure 5C).

Determination of the mtDNA/nDNA ratio by qPCR is regarded as an objective marker of the mtDNA copy number. According to previous studies, evaluation of the mtDNA copy number is a good indicator of the mitochondrial function (24). We measured mtDNA copy numbers in the groups and found that all four groups showed no statistically significant differences (Figure 5D). In summary, memantine, the NMDAR antagonist, prevented hypoxia-induced neonatal cardiac mitochondrial dysfunction.

### 3.5. Molecular Mechanism of Hypoxia-Induced Mitochondrial Dysfunction

#### 3.5.1. Hypoxia Could Induce the Elevation of the Glutamate Concentration Level in the H9C2 Cell Supernatant and Up-Regulation of NR1

**Figure 6:**
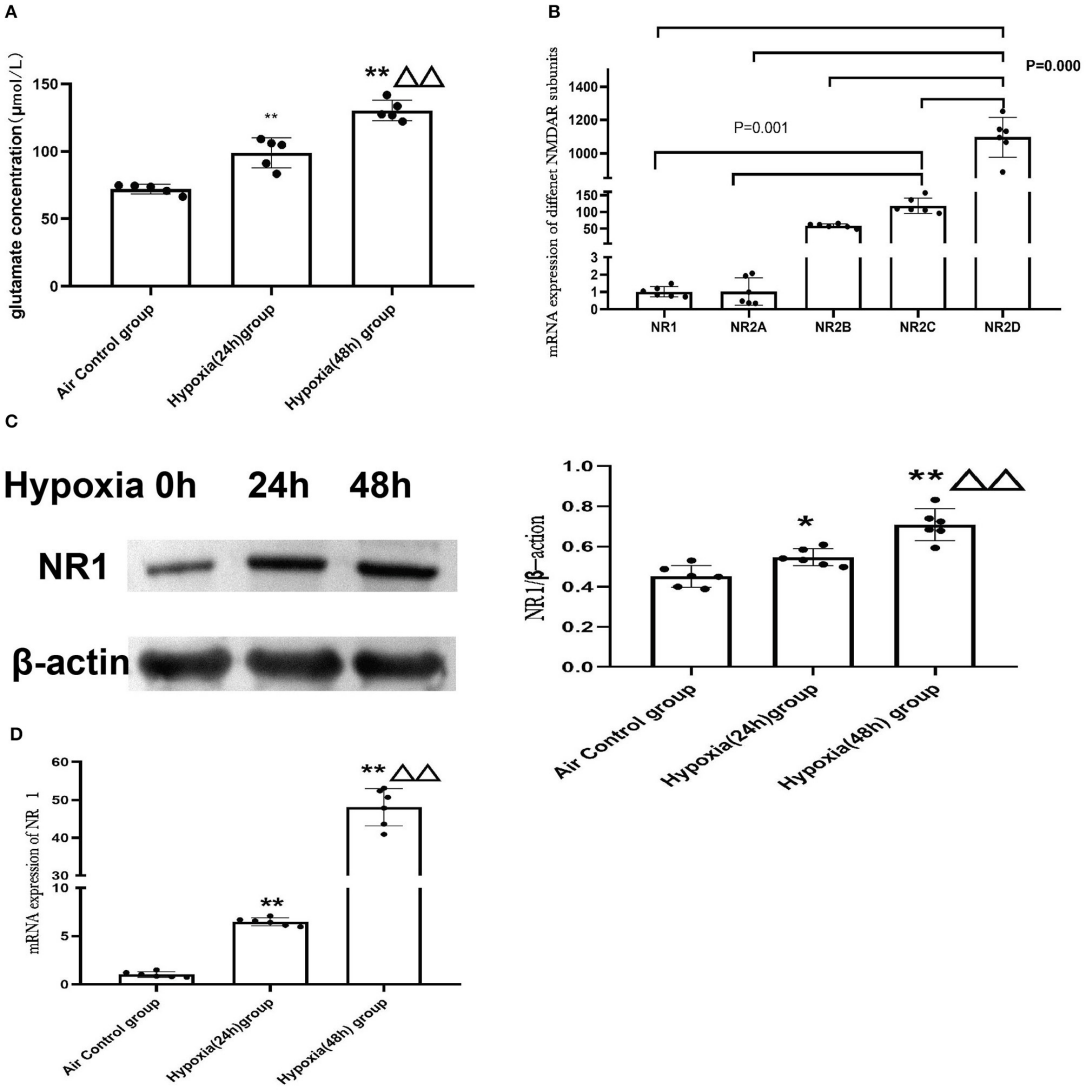
Hypoxia induced elevation of glutamate concentrations in H9c2 cell supernatant and increased the expression of NMDAR. **(A)** Glutamate concentration in the H9C2 cell supernatant (*N* = 5); **(B)** NMDAR subunit expression in H9c2 cells (*N* = 6); **(C)** The representative western blot images of NR1 after hypoxia exposure (**C**-left) and semi-quantified level of NR1 protein expression (**C**-right); **(D)** mRNA expression of NR1. Compared with the air control group: ^*^P < 0.05,^**^P < 0.01; compared with the Hypoxia 24 h group: ^▵^*P* < 0.05,^▵▵^*P* < 0.01. All data are presented as mean ± S.E.M.

To further explore the molecular mechanism of NMDAR in mitochondrial dysfunction, we used the cardiomyoblast cell line H9c2 to confirm and extend the *in vivo* results. Glutamate is the main excitatory neurotransmitter in the central nervous system that directly activates the NMDARs. We examined glutamate concentration in control cells (air) or cells exposed to hypoxia for 24 or 48 h. The data revealed that hypoxia led to increased glutamate levels and that with increased duration of hypoxia, the glutamate concentration was significantly increased (*P* < 0.01; Figure 6A). We then investigated the mRNA expression of the NMDAR subunits in H9c2 cells. We found that the distribution of NMDARs was similar to that in the animal model. The expression of NR2D was also the most abundant in the H9c2 cell line (Figure 6B).

In addition, we found that expression of the NR1 subunit was significantly upregulated after hypoxia treatment and demonstrated a time-dependent increase in both mRNAs (Figure 6D) and protein levels (Figure 6C).

#### 3.5.2. Excessive Activation of NMDAR Aggravated Mitochondrial Function *in vitro*

To explore the role of NMDAR activation in mitochondrial function, we incubated the cells with different concentrations of NMDA for 24 h and then assessed mitochondrial function. Cell viability following different durations and concentrations of NMDA treatment in H9cc2 cells is provided in the Supplementary Figure 4.

**Figure 7:**
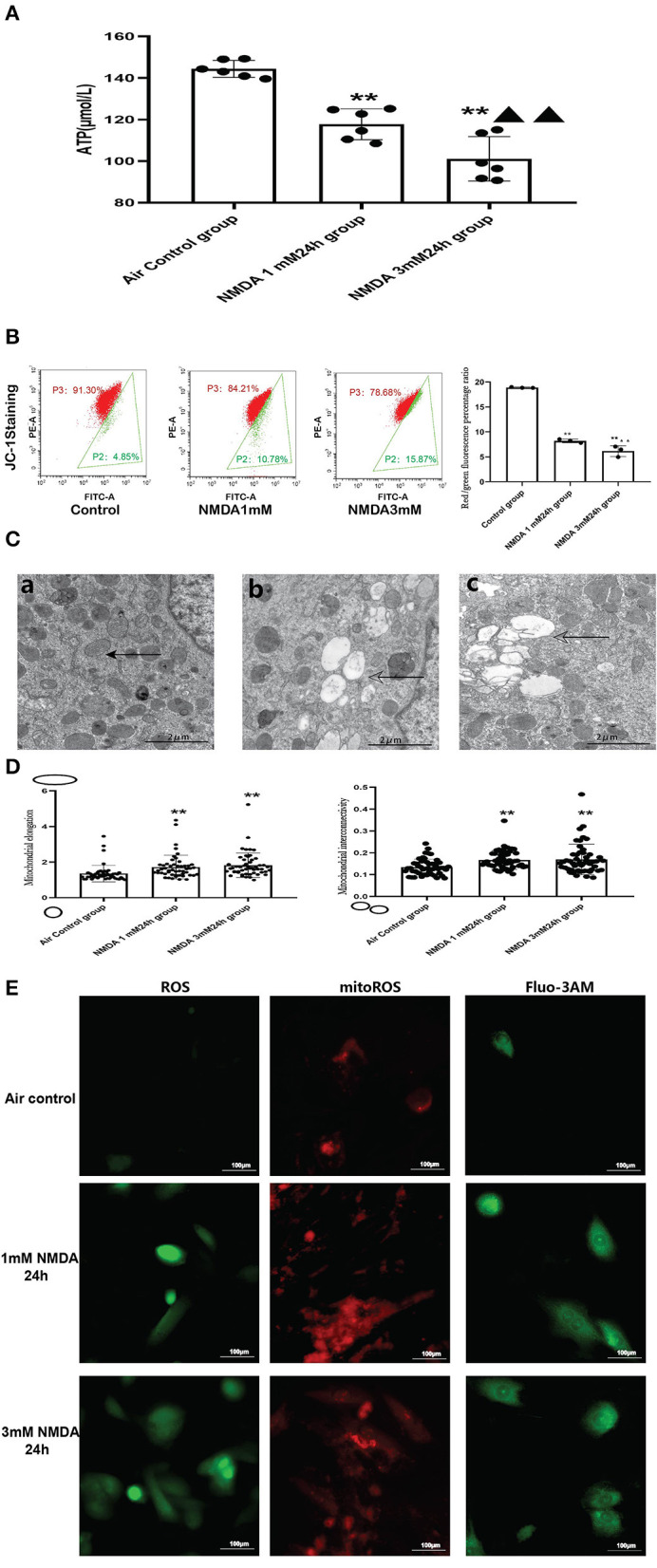
Excessive activation of NMDAR aggravated mitochondrial function. **(A)** Effects of NMDA exposure in ATP production (*N* = 6); **(B)** Effects of NMDA exposure in mitochondrial membrane potential; **(C)** Effects of NMDA exposure in mitochondrial ultrastructural damage (5000x), solid arrows: normal mitochondria; hollowed arrows: damaged mitochondria; (a): control group; (b): NMDA1mM group; (c): NMDA3mM group. **(D)** Effects of NMDA exposure in mitochondrial elongation (left) and interconnectivity (right); **(E)** Effects of NMDA exposure in ROS, mitoROS and intracellular calcium levels (400x); Compared with the air control group: ^*^P < 0.05,^**^P < 0.01; compared with the NMDA 1 mM 24 h group: ^▵^*P* < 0.05,^▵▵^*P* < 0.01. All data are presented as mean ± S.E.M.

We found that ATP levels decreased significantly in a dose-dependent manner after NMDA treatment, with higher concentrations causing lower ATP levels (Figure 7A).

When we examined the ultrastructure of the mitochondria, we found that NMDA incubation resulted in mitochondrial damage similar to what was seen in the animal model. There was significant mitochondrial vacuolation after NMDA treatment. The trend of mitochondrial elongation and interconnectivity was also similar to the animal model (Figures 7C,D). Our data revealed that NMDA incubation also significantly increased intracellular calcium levels (Figure 7E).

We also examined ROS/mitoROS and calcium levels following NMDA treatment of the cells. We found that NMDA treatment led to elevated ROS and mitoROS levels in the H9c2 cell line (Figure 7E). In order to explore whether the excess ROS production after NMDAR activation can induce mitochondrial damage, mitochondrial membrane potential was measured by using JC-1. Our flow cytometry analysis (Figure 7B) demonstrated the red/green fluorescence percentage ratio was significantly decreased, which indicated decreased MMP in the NMDA-treated cells compared to untreated cells (Figure 7B). The FSC vs SSC, FSC-A vs. FSC-H plots were provided in the Supplementary Figure 5. It was reported that the dysfunctions of the respiratory chain components, the decreased MMP and lower activity of the respiratory chain relusted in an increase in ROS production (25). The accumulated ROS would damage the mitochondrial membrane and change the mitochondrial membrane permeability, which would in turn reduce the mitochondrial membrane potential.

#### 3.5.3. Effects of Pharmacological Blockade of NMDARs or NR1 Knockdown on Mitochondrial Dysfunction

Our results concluded that the excessive activation of the NMDARs led to mitochondrial dysfunction *in vitro*. To further extend these results, we examined whether blocking NMDAR using either an antagonist or gene knockdown would alleviate the mitochondrial damage in response to hypoxia. MitoROS was used as a measure of mitochondrial function.

**Figure 8:**
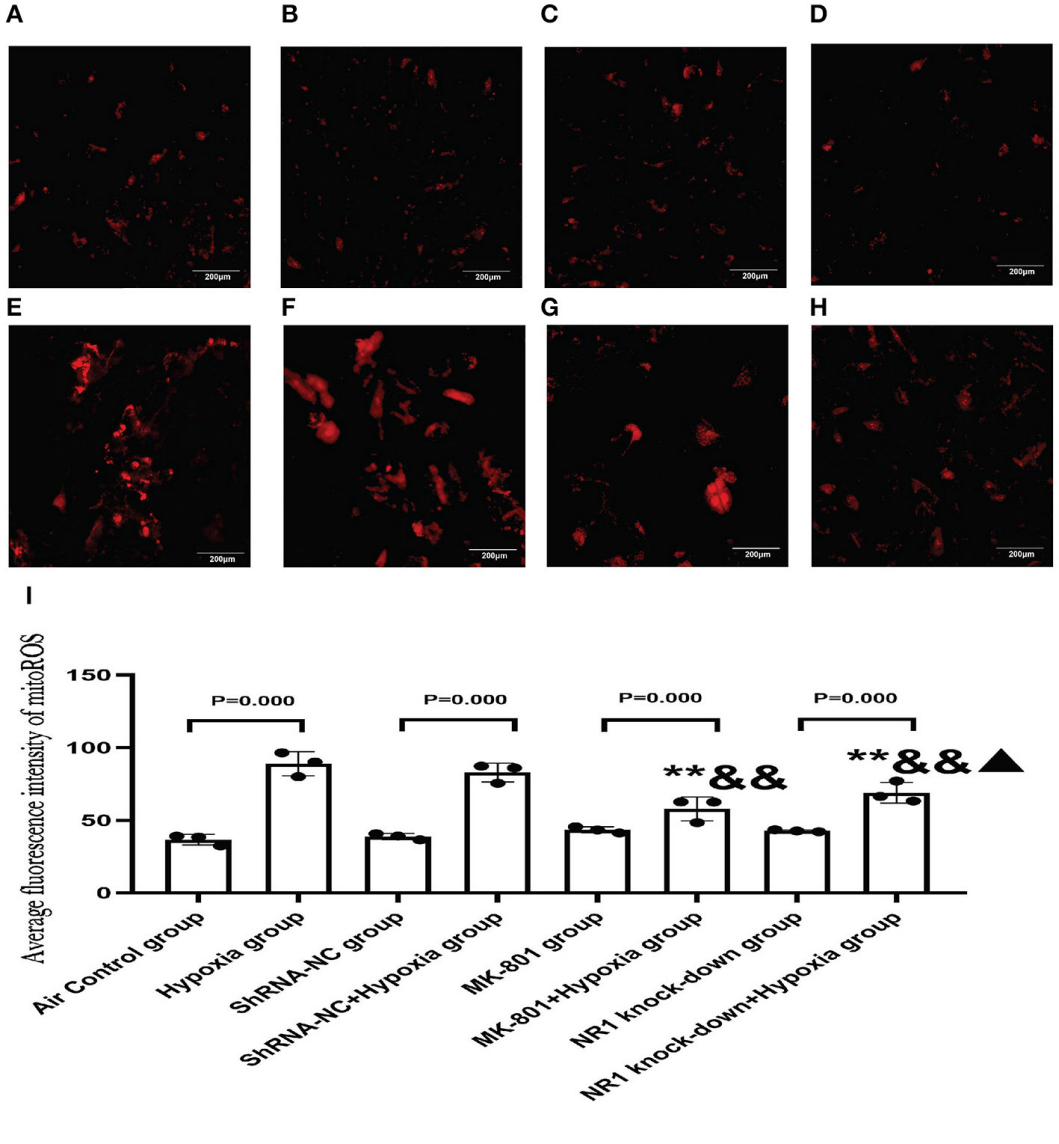
MitoSOX^*TM*^ Red staining for measuring mitoROS level. **(A–H)** The representative florescence images of MitoROS (200x). **(A–D)**: Air control group **(A)**, shRNA-NC group **(B)**, MK-801 group **(C)**, and NR1 knock-down group **(D)**; **(E–H)**: Hypoxia group **(E)**, shRNA-NC+hypoxia group **(F)**, MK801+hypoxia group **(G)**, and NR1 knock-down+hypoxia group **(H)**; **(I)** the average intensity of mitoROS fluorescence. Compared with the hypoxia group: ^*^P < 0.05,^**^P < 0.01; compared with the shRNA-NC+ hypoxia group: ^&^*P* < 0.05,^&&^*P* < 0.01; compared with the MK-801+hypoxia group: ^▴^*P* < 0.05,^▴▴^*P* < 0.01. All data are presented as mean ± S.E.M. (*N* = 3).

Our results revealed that MK-801 or NR1 knockdown alone showed on mitoROS in cells under normoxic conditions. Following hypoxia exposure, all four groups increased significantly compared to their respective controls (Hypoxia group vs. Air control group;shRNA-NC+Hypoxia group vs. shRNA-NC group; MK-801+Hypoxia group vs. MK-801 group; NR1 knock-down+Hypoxia group vs. NR1 knockdown group). However, MK-801 and NR1 knockdown alleviated the mitochondrial dysfunction induced by hypoxia, which reduced mitoROS levels. Mitoros levels in the MK-801+Hypoxia group and NR1 knock-down+ Hypoxia group were significantly lower than the Hypoxia group(*P* < 0.01). The mitoROS level of NR1 knockdown +hypoxia was the lowest among the four hypoxia-treated groups. In summary, MK-801 and NR1 knockdown alleviated the hypoxia-induced mitochondrial dysfunction by reducing the mitoROS level (Figure 8).

#### 3.5.4. Pharmarcological Blockage of NMDARs or NR1 Knock-Down Alleviated the Increase of Glutamate Concentration in Cell Supernatants and Calcium Overload

**Figure 9:**
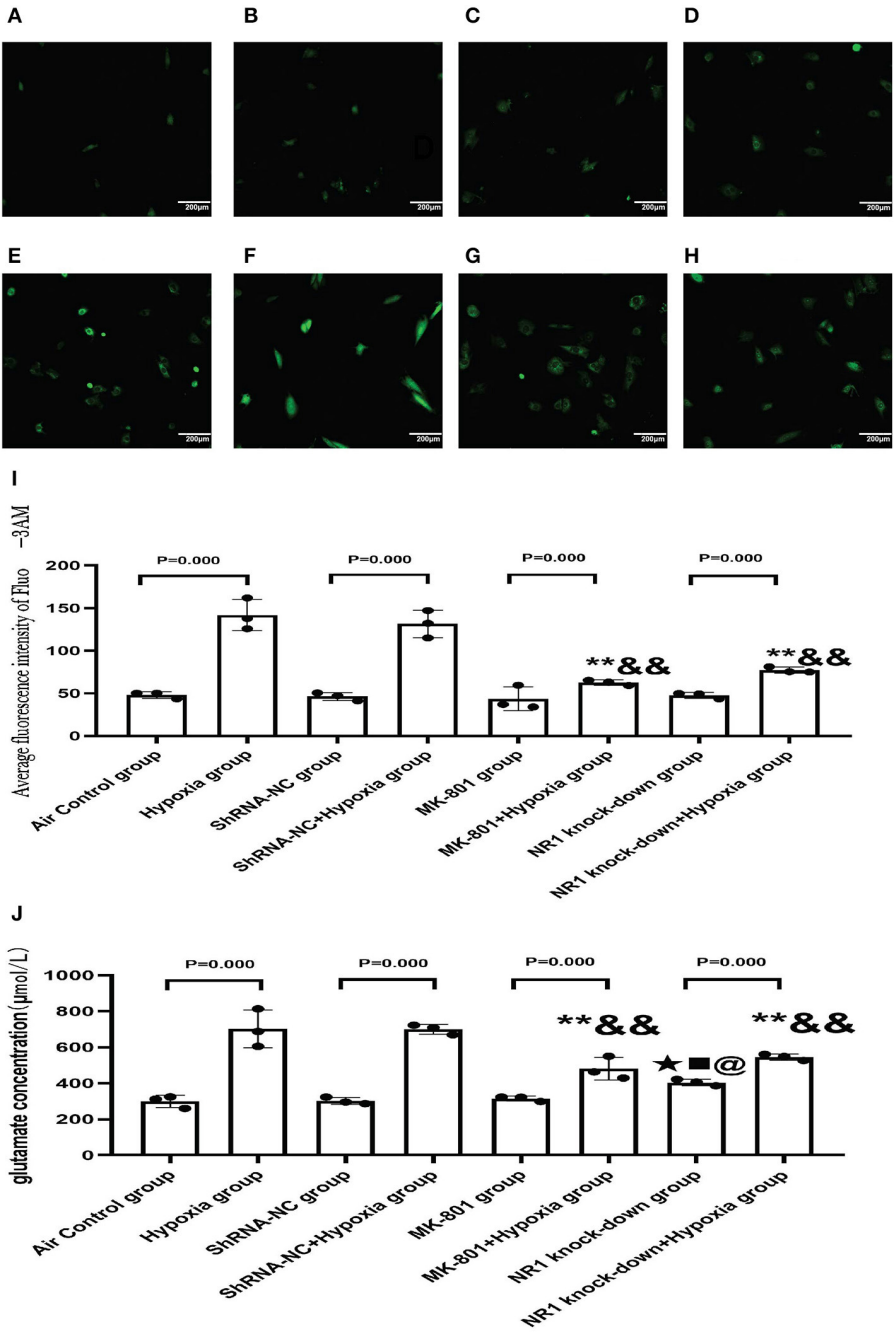
**(A–H)** The representative florescence images of Ca2+(200x). **(A–D)** Air control group **(A)**, shRNA-NC group **(B)**, MK-801 group **(C)**, and NR1 knock-down group **(D)**; **(E–H)**: Hypoxia group **(E)**, shRNA-NC+hypoxia group **(F)**, MK801+hypoxia group **(G)**, and NR1 knock-down+hypoxia group **(H)**; **(I)**: The average intensity of Ca2+ fluorescence; **(J)**: The glutamate concentration in different groups. Compared with the Hyppoxia group: ^*^P < 0.05,^**^P < 0.01; compared with the shRNA-NC+hypoxia group: ^&^*P* < 0.05,^&&^*P* < 0.01; compared with the air control group, ^▴^*P* < 0.05,^▴▴^*P* < 0.01; compared with the ShRNA-NC group, ^■^*P* < 0.05,^■■^*P* < 0.01;comparead with the MK-801 group, ^@^*P* < 0.05,^@@^*P* < 0.01. All data are presented as mean ± S.E.M. *N* = 3.

NMDARs function as a membrane calcium channel, and the activation of NMDARs leads to an influx of calcium ions, so we examined the intracellular calcium level in the present study (26). So we used NR1 knockdown and MK-801 treatment *in vitro* to explore the changes in intracellular calcium concentration and glutamate concentration after hypoxia exposure (24 h). Intracellular calcium level is an indication of NMDAR activation. MK-801 treatment and NR1 knockdown showed no effect under normoxic conditions. Following hypoxia treatment, significant calcium overload was induced in the control cells. However, pharmacological blockade of NMDARs and NR1 knockdown significantly attenuated the calcium overload induced by hypoxia, implying that the hypoxia-induced calcium overload was mediated through NMDAR signaling (Figures 9A–I). Glutamate could directly activate the NMDARs. Glutamate concentration in the NR1 knockdown group was significantly increased compared to the air control group. After hypoxia exposure, glutamate concentrations increased significantly in all four groups when compared to their respective controls (Hypoxia group vs. Air control group; shRNA-NC+Hypoxia group vs. shRNA-NC group; MK-801+Hypoxia group vs. MK-801 group; NR1 knock-down+Hypoxia group vs. NR1 knockdown group). In addition, we found that the glutamate concentration in the NR1 knockdown+Hypoxia group and MK-801+Hypoxia groups were reduced (*P* < 0.01 or 0.05) compared with the hypoxia group, which might indicate that NR1 knockdown and MK-801 incubation interfere with glutamate metabolism (Figure 9J).

#### 3.5.5. Intrauterine Hypoxia *in vivo* or NMDA Exposure *in vitro* Decreased Expression of DRP1

**Figure 10:**
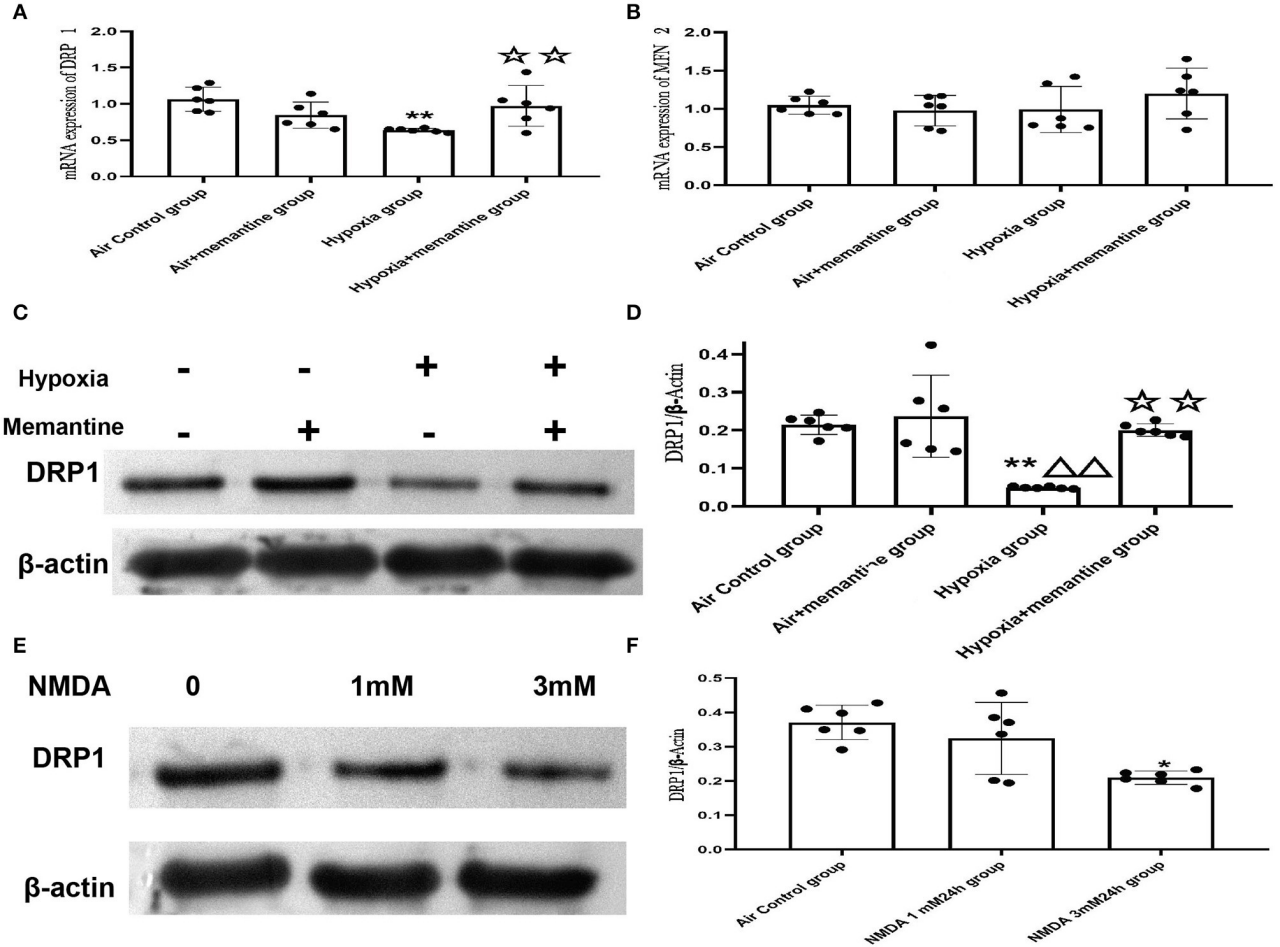
Intrauterine hypoxia *in vivo* or NMDA exposure *in vitro* decreased the expression of DRP1. **(A,B)** mRNA expression of DRP1 **(A)** and MFN2 **(B)**; **(C,D)**: The representative western blot images of DRP1 in IUH animal model **(C)** and semi-quantified level of DRP1 **(D)**; **(E,F)**: The representative western blot images of DRP1 after NMDA exposure **(E)** and semi-quantified level of DRP1 **(F)**; Compared with the air control group: ^*^P < 0.05,^**^P < 0.01; compared with the air+Memantine group: ^▵^*P* < 0.05,^▵▵^*P* < 0.01; compared with the hypoxia group: ^✰^*P* < 0.05,^✰✰^*P* < 0.01. All data are presented as mean ± S.E.M. (*N* = 6 *in vivo, N* = 3 *in vitro*).

Excessive activation of the NMDARs may be associated with the impaired cardiac mitochondrial function induced by antenatal hypoxic stress, but the underlying mechanism of the mitochondrial dysfunction remains unclear. The mitochondrion is a highly dynamic organelle, so it is reasonable to suspect that mitochondrial dynamics are damaged in the intrauterine hypoxia animal model or by NMDA exposure *in vitro*. Mitochondrial fission and fusion are the two major aspects of mitochondrial dynamics. Results from the mitochondrial ultrastructure studies *in vivo* and vitro were characterized by less mitochondrial fragmentation indicated more fusion or less fission. Mitofusin 2 (MFN2) and dynamin-related protein 1 (DRP1) are the critical fusion and fission regulators, respectively. We examined the mRNA expression of MFN2 and DRP1 in neonatal hearts of offspring from animals exposed to air or hypoxia. We found that the mRNA expression of MFN2 remained unchanged, suggesting that the mitochondrial fusion was not affected by intrauterine hypoxia on the first day of life (Figure 10B).

However, the results showed that DRP1 was downregulated by antenatal hypoxic stress, and antenatal blockade of NMDARs by memantine treatment significantly increased the expression of DRP1 (Figures 10A,C,D).

In the present study, antenatal memantine treatment significantly downregulated DRP1 expression. Thus, we assumed that DRP1 expression was associated with excessive activation of NMDARs. We examined DRP1 expression in H9c2 cells treated with NMDA, and the results validated that DRP1 protein expression was downregulated following treatment with 3 mM NMDA for 24 h (Figures 10E,F, *P* < 0.05). These results agree with those were seen *in vivo* and support the role of NMDAR activation in regulating mitochondrial dynamics.

## 4. Discussion

In the present study, we found that IUH-induced cardiac mitochondrial dysfunction was associated with increased activation of the cardiac NMDARs *in vivo*. By blocking NMDARs with memantine, the mitochondrial dysfunction improved in the IUH animal model. We confirmed the results *in vitro* using pharmacological blockade and genetic knockdown of NMDAR. Additionally, we demonstrated that the activation of NMDARs caused downregulation of DRP1 *in vitro*, which resulted in less fragmentation in mitochondrial morphology.

Antenatal hypoxia is a frequent complication during pregnancy and is also a cause of intrauterine growth retardation (27). Our results validated this by demonstrating that the BW and HW decreased. Additionally, the ratio of HW /BW increased. The HW/BW ratio increase may indicate that the effect of intrauterine hypoxia on BW and HW is asymmetric. It has also shown that when the fetus is under hypoxic stress, the blood vessels of vital organs such as the brain and heart dilate while those of other peripheral organs such as the lung and liver constrict, which leads to redistribution of blood flow. This redistribution is observed a few seconds after hypoxia (28). Fetus suffering from hypoxia experienced a decrease in blood supply to non-essential organs such as the lung and liver to ensure the blood and oxygen supply to vital organs such as the heart and brain, leading to an asymmetric growth restriction (29). In addition, The high pulmonary vascular resistance (PVR) associated with antenatal hypoxia causes afterload on the right heart leading to cardiac hypertrophy and insufficiency (30). Several studies have found that exposure to hypoxic stress during critical stages of development in chick and mammalian embryos (sheep, rabbits, rats, etc.) causes cardiac hypertrophy changes (31). Our results also confirmed the existence of cardiac hypertrophy by the WGA staining and the up-regulation of BNP in the neonatal offspring after intrauterine hypoxia.

According to Dr. Baker's fetal origins of adult disease hypothesis, intrauterine adverse stress, including prenatal hypoxia, significantly increases the incidence of cardiovascular disease in offspring (4). The heart is a highly dynamic organ and mitochondrial dysfunction is responsible for many cardiovascular diseases. Our study found that IUH during late pregnancy resulted in mitochondrial dysfunction characterized by reduced ATP levels and increased ROS levels in the neonatal rat heart tissue.CS activity is often regarded as a biomarker of the mitochondrial content and remained stable in the air+memantine group. From earlier research, memantine was proved to had no effect on CS activity *in vitro* (32). Our results are also consistent with Kim T Hellgren's research in a prenatal hypoxia mouse model (during gestational days 6–18). They found that male adult offspring from hypoxic pregnancies possess mitochondria with a reduced respiratory capacity and increased H_2_O_2_ production. However, their data mainly focused on the adult offspring aged 25–32 weeks which indicated that the mitochondrial damage could persist into adulthood (33). However, the underlying mechanism of impaired mitochondrial function is still controversial.

The main subunits of NMDARs are NR1 and NR2(2A-D). NMDA receptors are heterotetrameric assemblies of subunits. The classic NMDARs consist of two indispensable NR1 and two NR2. However, reports on the expression of the different subunits of the NMDARs in heart tissue are often conflicting. Joecelyn and their team found that only NR1 existed in the atrium and ventricle and that NR2 subunits were not expressed in the adult rat heart (34). On the contrary, another report demonstrated that NR2B, instead of NR1, could be detected from embryonic day 14 until postnatal day 21 but disappeared 10 weeks in the rat heart (35). The different results might be due to differences in timing, and location. We also demonstrated the existence of different NMDAR subunits including NR1 in newborn hearts and that NR2D was the most abundant subunit in the neonatal rat heart and H9c2 cell line, which is consistent with our previous study in the lung (36). But the actual function of NR2D in the cardiovascular system is undefined. The up-regulation of NR1 suggested being more vulnerable to the excitotoxic effects of endogenous glutamate (37). However, the focus of this study was to observe changes in NMDA receptors expression and glutamate concentrations, as these changes would increase susceptibility to glutamate-induced excitotoxicity. The underlying mechanism of the changes in NR1 and glutamate will be explored in our future study. Previous research demonstrated that ethanol increased protein kinase A (PKA) activation and cAMP-response-element-binding protein (CREB) phosphorylation, which increased N-methyl-D-aspartate receptor (NMDAR) expression (38). There is already a report about increased activity of PKA in hypoxia condition (39). In addition, memantine was proved to affect the PKA activity (40) and We then hypothesize that the increased NR1 after IUH may be due to the effect of hypoxia on the PKA/CREB signaling pathway.

Our team previously found that antenatal blockade of NMDA receptors by memantine reduced the susceptibility to diabetes induced by a high-fat diet after birth in rats in the same animal model (41). In the present study, prenatal treatment of memantine effectively attenuated the mitochondrial dysfunction as assessed by ATP and ROS levels. memantine treatment also significantly increased the CS activity in the hypoxia+memantine group, indicating more mitochondria in the treated group. This implies that the memantine could influence the mitochondrial content in the neonatal rat heart after hypoxia exposure during late pregnancy. A Previous study reported that NMDA receptor activation induces depolarized MMP in cultured neonatal rat cardiomyocytes (42). Another earlier report demonstrated that memantine decreased some oxidative stress biomarkers in the isolated rat heart (43). It indicated that the activation of NMDARs led to mitochondrial dysfunction *in vitro*. In the present study, NMDA exposure in H9c2 cells resulted in decreased ATP, MMP, and increased ROS and mitoROS levels providing further validation.

The impaired mitochondrial ultrastructure and function in offspring after intrauterine hypoxia and in cells following NMDA exposure were similar. Our data also demonstrated that prenatal memantine treatment effectively improved mitochondrial ultrastructure and function *in vivo*. It suggested that the activation of the NMDARs might be associated with the impaired mitochondrial function caused by antenatal hypoxia. Data *in vitro* revealed that the pharmacological blockade of NMDAR by MK801 or NR1 depletion could attenuate hypoxia-induced mitochondrial dysfunction *in vitro*. The glutamate levels in the cell supernatant after hypoxia exposure in the present study provided additional evidence that antenatal hypoxia over-activated the NMDARs. Similar results were reported in a study of myelination in brain development. Hypoxia insults during pregnancy led to the extracellular accumulation of excess glutamate in oligodendrocyte progenitor cells (44). Glutamate could directly activate the NMDARs. The NR1 knockdown *in vitro* could increase the glutamate concentration which indicated that the function of NMDAR could influence the glutamate secrete or transport. An intracellular calcium overload is a key event in the activation of NMDARs and we found that NR1 knockdown or pharmacological blockade of NMDARs effectively reduced increased calcium levels after hypoxia exposure. These findings directly indicate that hypoxia-induced mitochondrial dysfunction was mediated by excessive activation of NMDARs.

A previously published review concluded that mtDNA copy number control is an essential aspect of mitochondrial biogenesis (45). We found that the hypoxic stress did not affect the mtDNA copy number, which indicates that mitochondrial dysfunction in the animal model was not related to mitochondrial biogenesis. Our results also demonstrated that the morphology of the mitochondria in the IUH group or H9c2 cells after NMDA treatment was characterized by large vacuolated mitochondria with less mitochondrial fragmentation. Mitochondrial morphology and function are known to be closely connected. Mitochondrial function is modulated by its membrane fusion and fission. Longer, fused mitochondria are optimal for ATP generation, whereas fission of mitochondria facilitates mitophagy and cell division (46). The less mitochondrial fragmentation suggested that there might be less fission or more fusion in the present study. The imbalance between mitochondrial fission and fusion plays a vital role in mitochondrial dysfunction and cell senescence. In research concerning the aging process, the promotion of fusion or blockade of fission was an essential part of cell aging (47). We, therefore, investigated the expression of DRP1 and MFN2 in the animal model and H9c2 cell line. Expression of the essential mitochondrial fusion gene MFN2 did not show any difference which indicated the mitochondrial fusion was not damaged.

DRP1 is a crucial regulator of mitochondrial fission (48), and DPR1 was significantly downregulated in the IUH group and NMDA-exposed H9c2 cell line in the present study. In the previous study about the hyperoxia-induced bronchopulmonary dysplasia animal model, the expression and phosphorylation of Drp1 increased in lung tissues. When Drp1 expression was inhibited, the small pulmonary vessel development improved, and pulmonary hypertension was relieved (49). DRP1 also plays a vital role in heart development, and DRP1 knockout mice exhibited fatal cardiac defects (50). All mice died between day 9 to 11 and echocardiography at postnatal day 7 demonstrated significantly reduced left ventricular function (50). In addition, the mitochondrial morphology in DRP1 knockout mice showed enlarged mitochondria with large vacuolar structures that contained remnants of mitochondria (50), which were quite similar to that seen in our animal model. Based on these results, depletion of DRP1 could interfere with heart development and inhibit mitochondrial fission damaging the mitochondrial dynamics. Interestingly, another study found that the longer intrauterine hypoxia (G15-21) could lead to up-regulation of DRP1, more mitochondrial fragments, and impaired mitochondrial function in offspring's heart at postnatal day 7. The differing results could be explained by changes in the duration, the timing of the prenatal hypoxic stress, and the stage of postnatal heart development, which requires more research (6). It is usually accepted by us that decreased mitochondrial fusion and up-regulated mitochondrial fission were deleterious, while increasing mitochondrial fusion and decreasing mitochondrial fission were beneficial. However, the recent studies challenged this idea (51, 52). Mitochondria of Drp1 depletion were bigger and demonstrated mitochondrial dysfunction (51). The DRP1 knockdown could significantly result in muscle atrophy and remodeling (52) and severe muscle wasting, weakness, and degeneration (51). However, the present study didn't clarify the mechanism of down-regulated DRP1 after intrauterine hypoxia. The Hypoxia inducible factor (HIF) is a transcription factor synchronizing the hypoxic environment and widely investigated in the study of cancer and hypoxia signaling pathway (53–55). The HIF-1 was reported to be associated with the expression of DRP1 (56). Thus, we will explore the HIF-1 expression in the future study.

Inevitably, our study had some limitations. Importantly, our experiments found decreased expression of DRP1, but the relationship between DRP1 and mitochondrial dysfunction is still unclear. The underlying mechanism still needs further research. In addition, we used mitoROS to assess the mitochondrial function, which might not be accurate for the artifacts of the dye. We would use other live-cell mitochondrial function assessments, including the Oxygen consumption rate (OCR), in the future to minimize the artifactual errors.

## 5. Conclusion

IUH resulted in declining birth weight, heart weight, and an abnormally high heart weight/birth weight ratio. Furthermore, IUH caused mitochondrial structural, functional abnormalities, and decreased expression of DRP1, and upregulation of NMDAR1 *in vivo*. Antenatal memantine treatment improved these changes. *in vitro*, hypoxia increased the glutamate concentration and expression of NMDAR1. NMDAR activation may lead to similar changes in mitochondrial function, structure, and downregulation of DRP1 *in vitro*. Pharmacological blockade of NMDARs by the non-competitive NMDA antagonist MK-801 or knockdown of the glutamate receptor NR1 significantly attenuated the increased mitochondrial reactive oxygen species and calcium overload-induced by hypoxia exposure. In summary, the present study demonstrated that neonatal cardiac mitochondrial dysfunction induced by intrauterine hypoxia might be associated with excessive activation of NMDARs. The underlying mechanism of IUH-induced mitochondrial damage might be associated with the downregulation of DRP1, which was also mediated by NMDARs. Memantine's effective attenuation of the impaired cardiac mitochondrial function seen in this study promises future investigation of its clinical application in prenatal hypoxia. The mechanism of the up-regulation of NR1 and the down-regulated DRP1 after IUH are the focus of our future research.

## Data Availability Statement

The original contributions presented in the study are included in the article/Supplementary Materials, further inquiries can be directed to the corresponding author.

## Ethics Statement

The animal study was reviewed and approved by the Ethics Committee of Central South University, Department of laboratory animals (protocol code 2018sydw001 and date of 2017.12.27).

## Author Contributions

SY and ZQL: conceptualization. YL and TL: methodology. YL: formal analysis and investigation. YL and CC: writing original draft preparation. YZ, ZCL, and SL: writing review and editing. SY: supervision. MW: project administration. ZCL and YD: funding acquisition. All authors have read and agreed to the published version of the manuscript.

## Funding

This research was funded by National Natural Science Foundation of China (grant nos. 82071693 and 81801510) and Hunan Province Natural Science Foundation of China (grant nos. 2019JJ50930 and 2021JJ31046).

## Conflict of Interest

The authors declare that the research was conducted in the absence of any commercial or financial relationships that could be construed as a potential conflict of interest.

## Publisher's Note

All claims expressed in this article are solely those of the authors and do not necessarily represent those of their affiliated organizations, or those of the publisher, the editors and the reviewers. Any product that may be evaluated in this article, or claim that may be made by its manufacturer, is not guaranteed or endorsed by the publisher.
