# Supplementary material for: Effects of Excessive Activation of N-methyl-D-aspartic Acid Receptors in Neonatal Cardiac Mitochondrial Dysfunction Induced by Intrauterine Hypoxia
Source: Front Cardiovasc Med. 2022 Mar 30;9:837142. doi: 10.3389/fcvm.2022.837142 (PMC9039344; doi:10.3389/fcvm.2022.837142)
Supplement: Supplementary file 1 [file Data_Sheet_1.pdf]

Supplementary materials

Figure S1. The NR1 siRNA validation by western blot. 1.control group;2.shRNA-NC group;3.NR1 siRNA-1 group;4.NR1 siRNA-2 group;5.NMDAR1 siRNA-3 group.6.Brain tissue(positive control).It is obvious that NR1 siRNA-1 is most efficient.

Figure S2. The representative images of immunofluorescence of NR1(red) and CTnT (green) in newborn rat heart (200x and 400X).

FigureS3.The mRNA expression of NR2D in vivo. Compared with the air control group: *,P<0.05.

Figure S4. Cell viability of different duration and concentration of NMDA in H9C2 cells.
Compared with the control group: *,P<0.05, **,P<0.01.

Figure S5. The FSC vs SSC, FSC-A vs FSC-H plots of the JC-1.

FigureS6. The graph abstract of the present study.

TableS1. The animal group information for the present study.

TableS2.The Primer information for the present study.

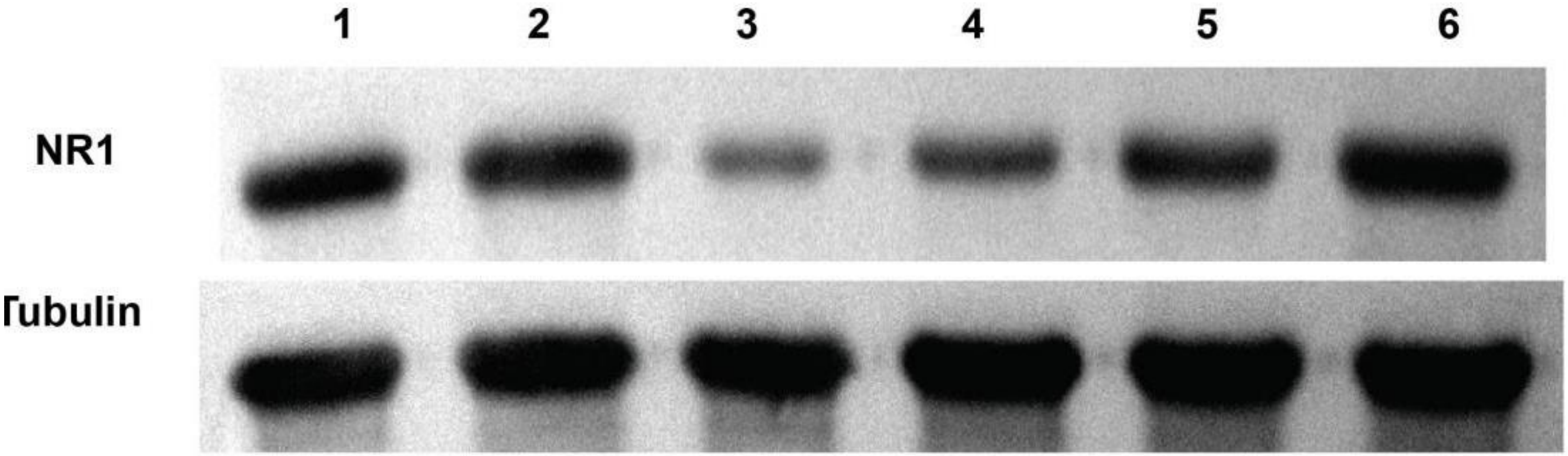

Figure S1

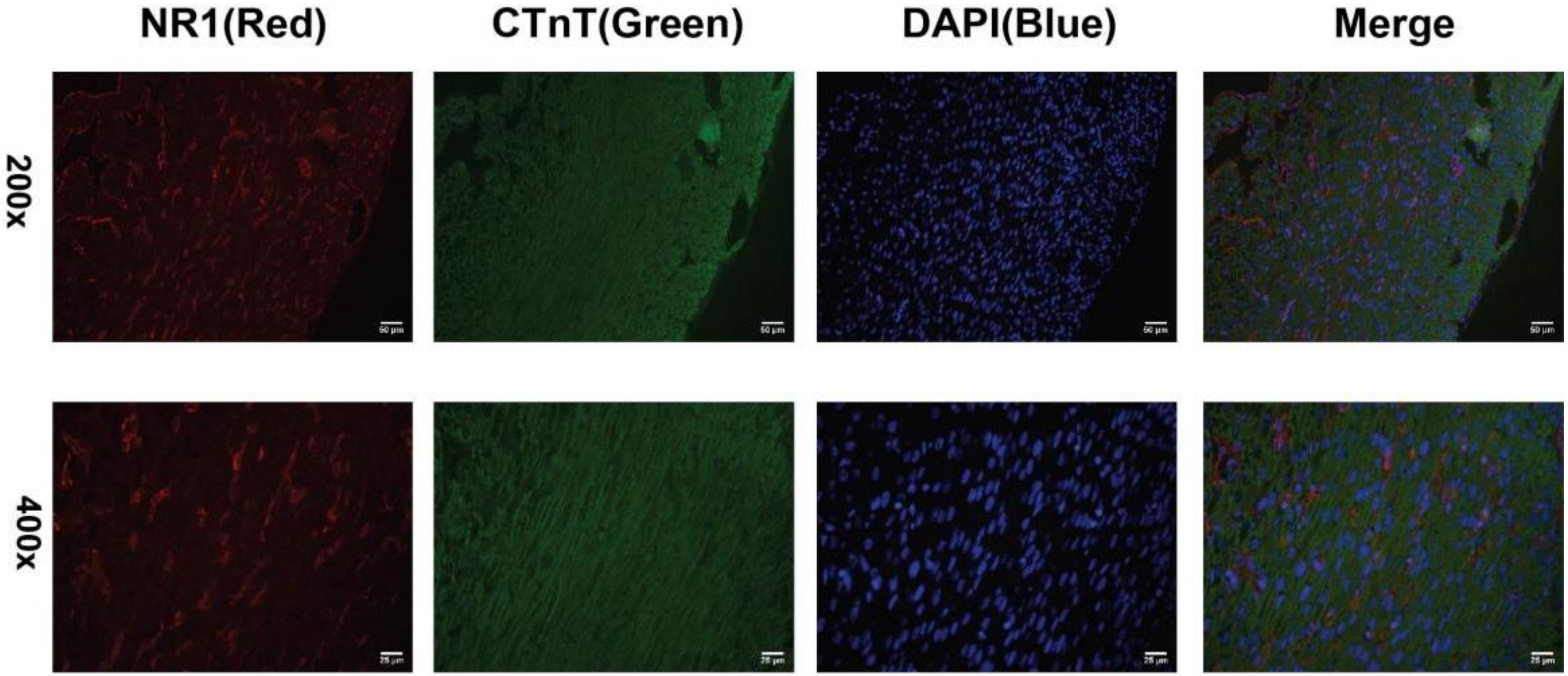

Figure S2

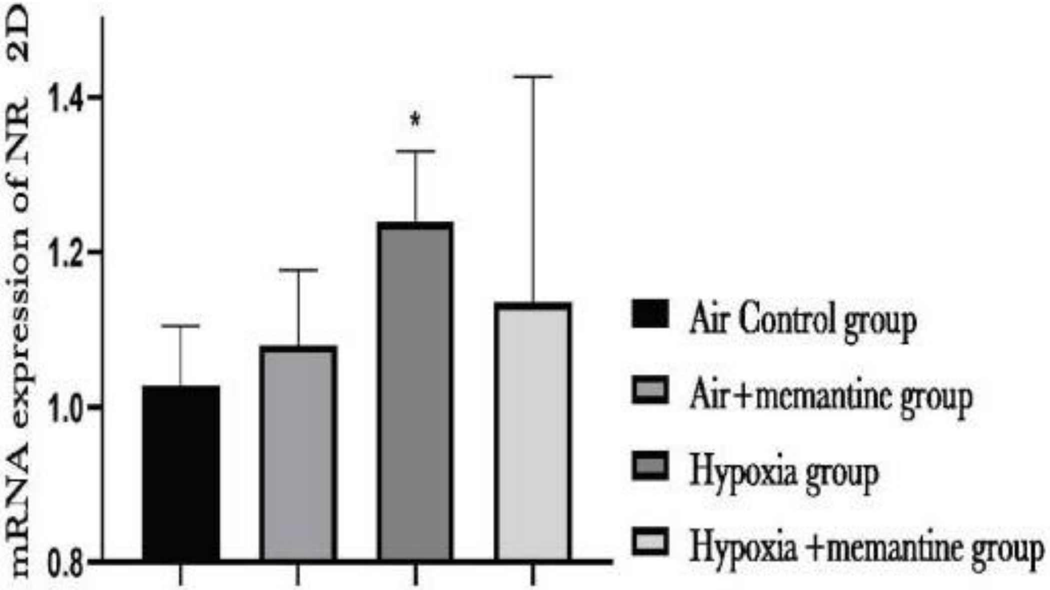

Figure S3

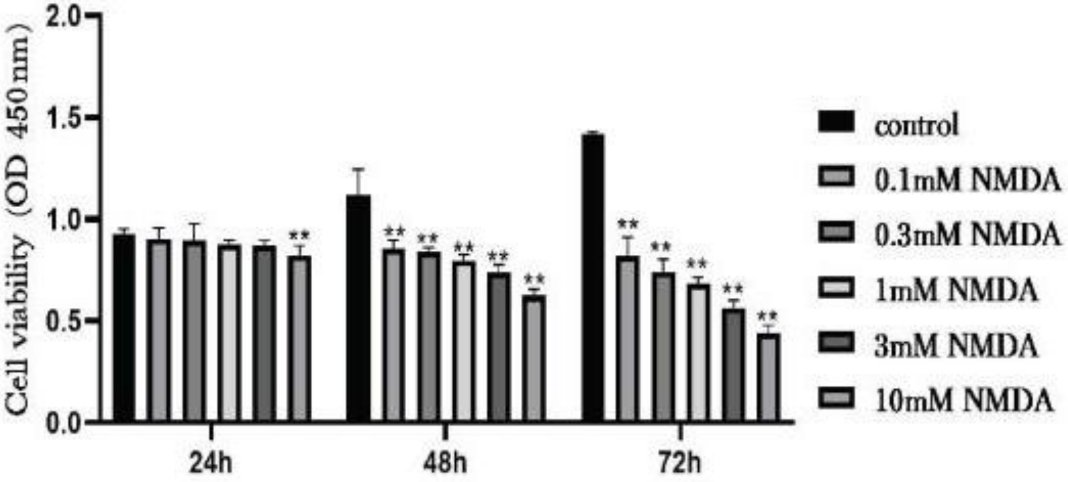

Figure S4

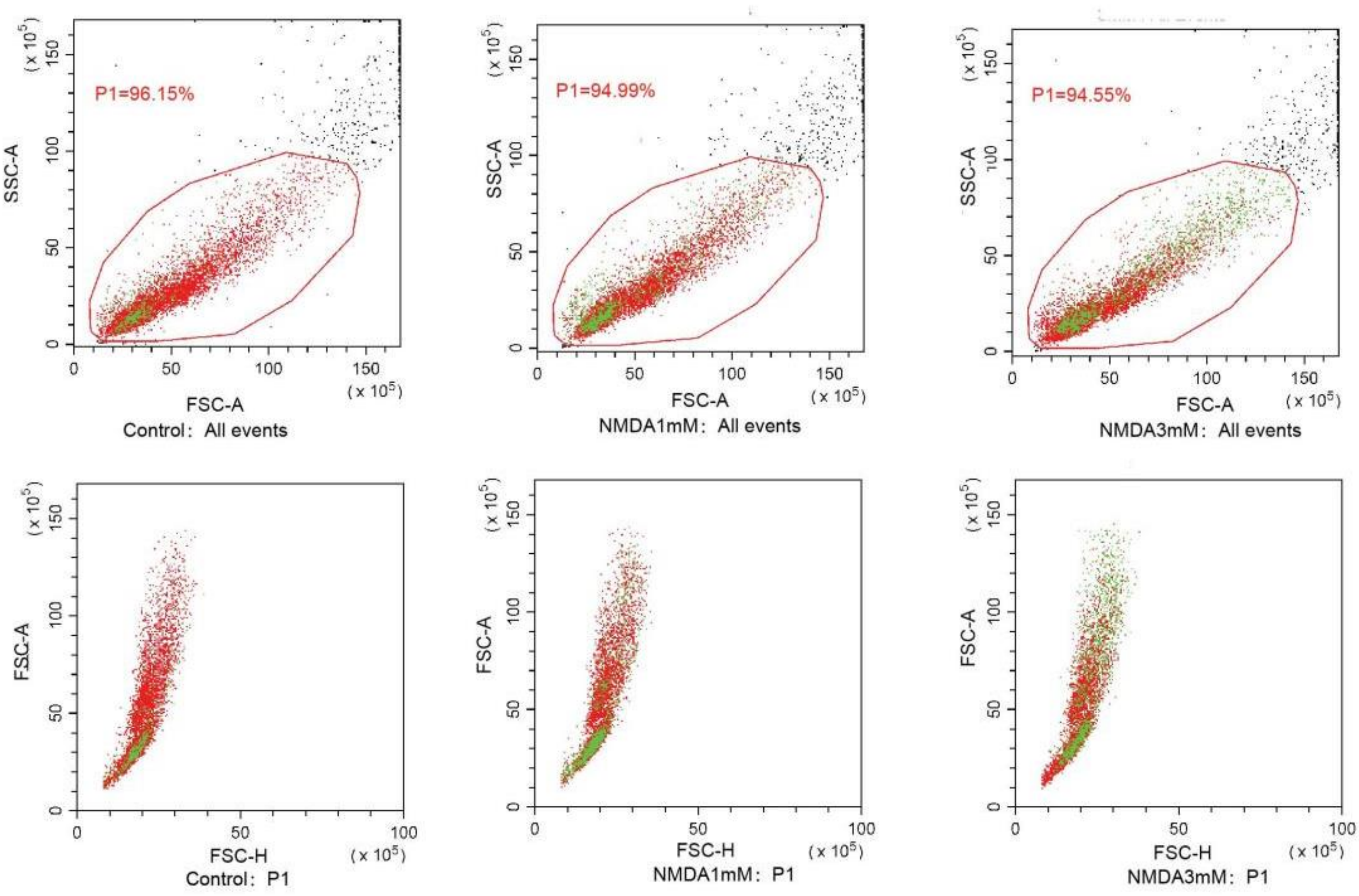

P1=96.15%
SSC-A
(x 10⁵)
FSC-A
Control: All events
P1=94.99%
NMDA1mM: All events
P1=94.55%
NMDA3mM: All events
FSC-H
Control: P1
NMDA1mM: P1
NMDA3mM: P1

Figure S5

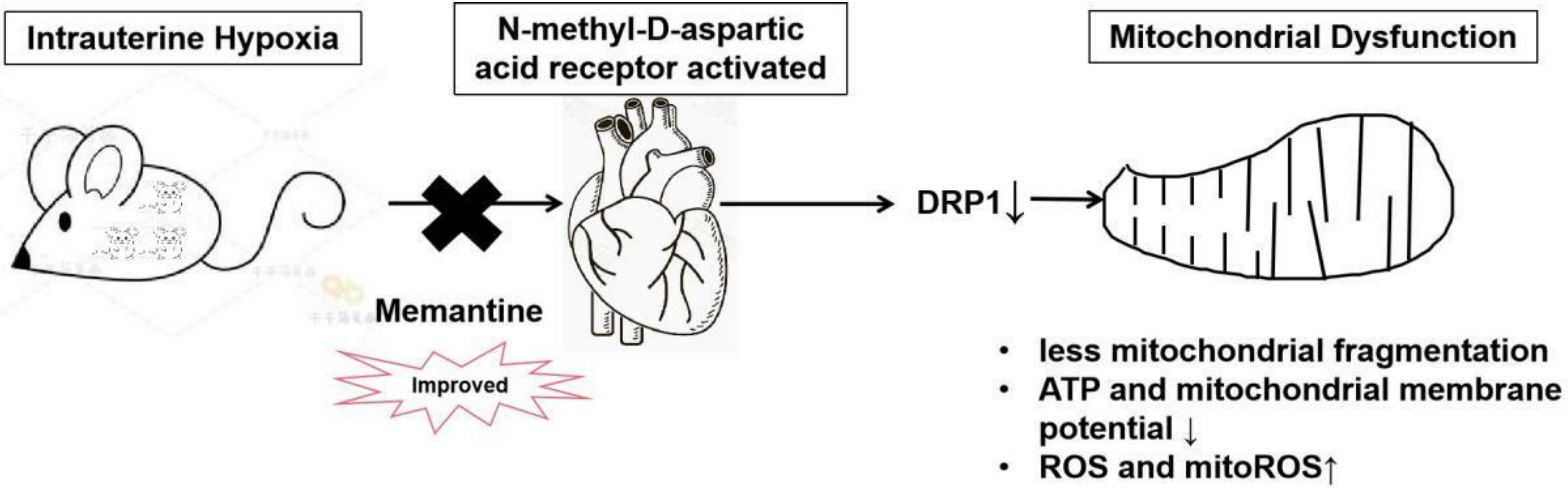
Intrauterine Hypoxia
N-methyl-D-aspartic acid receptor activated
Mitochondrial Dysfunction
Memantine
Improved
DRP1↓
less mitochondrial fragmentation
ATP and mitochondrial membrane potential ↓
ROS and mitoROS↑

Figure S6

Table S1. The animal group information for the present study.

Group	Treatment time	Treatment
Air control group	G19-20	21% O2 (Air)
Air+mematine group		21% O2 (Air)+memantine intraperitonal(5mg/kg/d，Qd)
Hypoxia group		9.5 to 11.5% O2（Hypoxia）for 8 hours per day
Hypoxia+memantine group		9.5 to 11.5% O2（Hypoxia）for 8 hours per day+memantine intraperitonal(5mg/kg/d，Qd)

Table S2. The Primer information for the present study.

Gene name	forward	reverse
DRP1	5′-ACAACAGGAGAAGAAAATGGAGT-3′	5′-CGTTGGGCGAGAAAACCTTG-3′
NMDAR1	5′-CCTACTCCCAACGACCACT-3′	5′-CACCGTGCGAAGGAAACTCA-3′
MFN2	5′-CTCAGGAGCAGCGGGTTTATTGTCT-3′	5′-TGTCGAGGGACCAGCATGTCTATCT-3′
