# Supplementary material for: Effects of Excessive Activation of N-methyl-D-aspartic Acid Receptors in Neonatal Cardiac Mitochondrial Dysfunction Induced by Intrauterine Hypoxia
Source: Front Cardiovasc Med. 2022 Mar 30;9:837142. doi: 10.3389/fcvm.2022.837142 (PMC9039344; doi:10.3389/fcvm.2022.837142)
Supplement: Supplementary file 2 [file Data_Sheet_2.pdf]

Figure 2: https://www.jianguoyun.com/p/DanR9uwQlNn1CRidrpQE

Figure 3: https://www.jianguoyun.com/p/DXXXWMwQlNn1CRiorpQE

Figure 4: https://www.jianguoyun.com/p/DTBoy_kQlNn1CRiqrpQE

Figure 6: https://www.jianguoyun.com/p/DeR0BHwQlNn1CRixrpQE

Figure 7: https://www.jianguoyun.com/p/DQ5MHlEQlNn1CRizrpQE

Figure 8: https://www.jianguoyun.com/p/Dd8YOZwQlNn1CRi1rpQE

Figure 9: https://www.jianguoyun.com/p/DY0L8cUQlNn1CRi9rpQE

Figure 10: https://www.jianguoyun.com/p/DfR92X8QlNn1CRjArpQE

Figure S1: https://www.jianguoyun.com/p/DcDPgYEQlNn1CRjCrpQE

Figure S2: https://www.jianguoyun.com/p/DYkDFfsQlNn1CRj-kK4E
